# *EHP* Paper of the Year, 2010

**DOI:** 10.1289/ehp.1002330

**Published:** 2010-06

**Authors:** Hugh A. Tilson

**Affiliations:** E-mail: tilsonha@niehs.nih.gov

The Paper of the Year—established by *Environmental Health Perspectives* (*EHP*) as a means of reinforcing high-quality articles published in the journal, identifying emerging research themes, and tracking the impact of groundbreaking research (Tilson 2008)—is selected by tracking papers published in *EHP* during the preceding 60 months that have been cited most frequently in the environmental health sciences literature. In this issue, we are pleased to announce that the *EHP* Paper of the Year for 2010 is “A Toxicologic Review of Quantum Dots: Toxicity Depends on Physicochemical and Environmental Factors” by Ron Hardman (2006).

At the time this paper was published, little attention had been given to quantum dot technology from a toxicologic perspective, and most nanotoxicology papers and conferences had focused on carbon-based nanomaterials, with lesser emphasis on metal- and metalloid-based nanomaterials. For many readers this paper may have been their first real exposure to quantum dot technology, the societal benefits that may be afforded by the technology (e.g., transistors, solar cells, next-generation flat panel displays, biosensing devices, medical imaging, and quantum computing), and the potential adverse environmental and human health effects that may result from the manufacture, use, and disposal of quantum dots.

This review article addressed a novel technology and synthesized what was currently known about these materials. The paper drew attention to the fine balance between a technologic advancement and the potential adverse societal impacts of such an advance when its implementation is not well understood. History has shown that technologic advances have often had deleterious impacts on human health and the environment (e.g., leaded gasoline, polychlorinated biphenyls, polybrominated diphenyl ethers, perfluorochemicals). It was not so much the technologies themselves that had direct deleterious effects on humans and ecosystems, but rather our ignorance of how to wisely use these technologies and products commercially.

Central to the issue of emerging nanotechnologies—including quantum dots—is the fact that environmental problems often have a significant economic basis. The majority of harmful substances found in our environment are by-products of economic activity. Quantum dot technologies, due to their significant potential as drivers of economic growth, may pose significant risks to the environment and human health if introduced into the commercial marketplace without due consideration of their potentially deleterious effects.

There is much we simply do not know in regard to the environmental transport, fate, and potential human health effects of these materials. Hence, the challenge is how to wisely and creatively use these technologies, minimizing or precluding exposure and risk, keeping in mind that the relationships among the environment, our economic endeavors, and human health are deeply integrated.

*EHP* congratulates Dr. Hardman for his contribution to the environmental health science literature. His paper (Hardman 2006) drew attention to the emerging field of quantum dot technology, and expressed to the public and scientific community issues regarding the benefits, risks, and wise development and application of this novel technology.

## Figures and Tables

**Figure:**
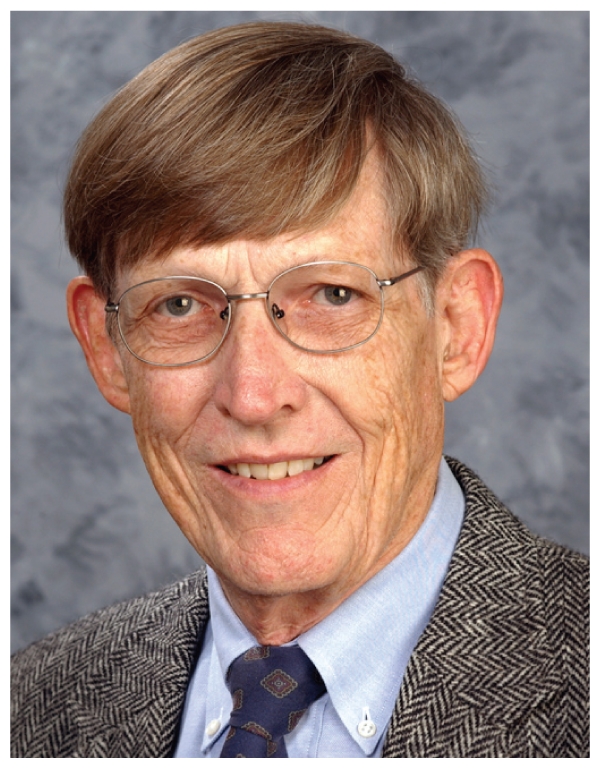
Hugh A. Tilson
